# Maternal imprinting of the neonatal microbiota colonization in intrauterine growth restricted piglets: a review

**DOI:** 10.1186/s40104-019-0397-7

**Published:** 2019-11-11

**Authors:** Lili Jiang, Cuiping Feng, Shiyu Tao, Na Li, Bin Zuo, Dandan Han, Junjun Wang

**Affiliations:** 1 State Key Laboratory of Animal Nutrition, College of Animal Science and Technology, China Agricultural University, Beijing, 100193 China; 2 Department of Obstetrics and Gynecology, China-Japan Friendship Hospital, Beijing, 100029 China

**Keywords:** IUGR piglet, Microbial colonization, Maternal imprinting, Nutritional intervention

## Abstract

Early colonization of intestinal microbiota during the neonatal stage plays an important role on the development of intestinal immune system and nutrients absorption of the host. Compared to the normal birth weight (NBW) piglets, intrauterine growth restricted (IUGR) piglets have a different intestinal microbiota during their early life, which is related to maternal imprinting on intestinal microbial succession during gestation, at birth and via suckling. Imbalanced allocation of limited nutrients among fetuses during gestation could be one of the main causes for impaired intestinal development and microbiota colonization in neonatal IUGR piglets. In this review, we summarized the potential impact of maternal imprinting on the colonization of the intestinal microbiota in IUGR piglets, including maternal undernutrition, imbalanced allocation of nutrients among fetuses, as well as vertical microbial transmission from mother to offspring during gestation and lactation. At the same time, we give information about the current maternal nutritional strategies (mainly breastfeeding, probiotics and prebiotics) to help colonization of the advantageous intestinal microbiota for IUGR piglets.

## Introduction

The gastrointestinal tract (GIT) of mammalian animals contains a large microbial community [1]. Early colonization of the intestinal microbiota is believed to be paramount for maturation of the intestinal innate immune system and barrier function, as well as health of the host [2, 3]. At the same time, the intestinal microbiota in neonates is extremely turbulent and can be shaped by the different physiological status of their host [4], the dietary changes [5], and the feeding environments [6, 7]. A recent study has indicated that the intestinal microbiota of IUGR piglets was significantly different from that of the NBW piglets during their neonatal stages [8]. Considering the delivery transition from relative sterile environment in uterus to the complex bacterial environment in farrowing house, the impaired small intestine of newborn IUGR piglets could be a starting point for the postnatal dysbiosis of intestinal microbial community. Therefore, the microbiota colonization in IUGR piglets could be maternally imprinted, due to malnutrition of sows or imbalanced allocation of limited nutrients among fetuses during gestation.

Given these developmental deficits of the intestine and their microbiota in IUGR piglets, the purpose of this review article is to review the potential ways from the perspective of maternal imprinting. As well, the nutritional strategies for improving colonization of the advantageous intestinal microbiota in neonatal IUGR piglets are also summarized, with a perspective of maternal intervention.

### Maternal malnutrition as a reason for occurrence of the IUGR piglets

During the mid and late gestation, the utero-placental circulation and umbilical cord vein are mainly responsible for delivering the nutrients from the mother to the fetuses [9]. It has been reported that the transportation of nutrients from mother to IUGR porcine fetuses was altered during gestation due to the decreased blood flow in placenta [10, 11]. Expression of several proteins related to energy metabolism was decreased in placenta and endometrium of the IUGR fetuses (d 60, 90, and 110 of gestation), which could contribute to the inadequate energy provision and insufficient nutrient transport and thus the occurrence of IUGR [12]. One important feature was the insufficient amino acid transmission from the sow to the IUGR fetuses [13]. Specifically, IUGR fetus had a decreased supply of amino acids in the arginine family such as arginine and glutamine, and also the branched chain amino acids (valine, leucine, and isoleucine), as well as glucose, while increased levels of ammonia in the umbilical cord vein [14]. In an obese sow model, maternal malnutrition (50% standard grain-based diet) during the last two-thirds of gestation induced asymmetryical growth retardation and metabolic alterations in the newborn piglets [15]. In addition, Mickiewicz et al. [16] and Metges et al. [17] found that low protein diets (6.5% protein) administrated to gilts led to IUGR, and even the delayed catch-up growth in IUGR piglets, it was possibly a lack of indispensable amino acids that led to injured lipoprotein metabolism. Likewise, feeding a low-protein diet (50% of standard-protein) to the sows during late-gestation resulted in notable decrease in birth weight of newborn piglets, as well as the reduced expression and activity of 11β-hydroxysteroid dehydrogenase 2 in placenta with a sex-dependent way [18].

Oocyte maturity might be a crucial factor of embryonic uniformity and subsequent within-litter variation in birth weight [19], therefore, nutritional supplies during pre-mating or peri-implantation period may have significant effects on within-litter uniformity of the birth weight. Large numbers of evidences have suggested that the maternal malnutrition before breeding and the peri-implantation period posed a threat on the oocyte quality and embryonic development [20, 21]. Feeding low-energy diets to sows during the weaning-to-estrus interval lowered ovulation rate, follicle size and litter homogeneity [22]. While appropriate increasing energy intake (3.5 kg/d) for pre-mating sows can decrease the within-litter variability in blastocyst size at d 12 of pregnancy, compared with that from sows fed a maintenance diet (1.15 kg/d) [23]. Moreover, the uniformity of birth weight in the litter was decreased in sows on dextrose-supplemented diets (150 g/d) compared to the sows fed basal-diet during the weaning-to-estrus interval [24]. Therefore, modest energy requirements for sows prior to mating have a crucial impact on within-litter uniformity.

### The developmental defects in the intestine of IUGR piglets

Recent studies identified an impairment of intestinal development in IUGR piglets at birth [25, 26], and this injury persisted during the whole suckling period [27, 28]. One of the causes of this damage was the abnormally regulated DNA methylation [29, 30]. As well, the intestinal barrier integrity were injured in the IUGR newborn piglets, demonstrated as damaged villi, shorter microvilli, reduced villus surface areas, fewer number of epithelial goblet cells or lymphocyte, and the decreased levels of the cytokines such as tumor necrosis factor-α and interferon-γ as well as their gene expressions [31]. Additionally, the decreased intestinal immunity function in IUGR piglets was connected with overexpression of the heat shock protein 70, which impairs the nuclear factor-kappa B signaling and upregulates forkhead box O3a expression in the intestine [32]. One of the possible mechanisms was targeted degradation of the proteins in tight junction pathways and extracellular matrix by the miRNA-29a, which then results in the impairment of intestinal epithelial integrity [33]. Taken all together, the developmental defects in the intestine and intestinal immune system of IUGR piglets are mainly mediated by changes in the key cytokines, immune-related proteins and inflammation-related cell signaling pathways, thus resulting in poor nutritional absorption and high risk of intestinal infection, as well as the higher morbidity and mortality in their early postnatal life.

### The altered intestinal microbiota in neonatal IUGR piglets

Accompanying the injured intestinal barriers in IUGR piglets, the establishment and succession of their intestinal microbiota is also changed. A previous study found that the permeability of macromolecules through the intestinal barrier of IUGR piglets was increased [34], leading to higher counts of adherent bacteria to the intestinal mucosa [35, 36]. Recent research has suggested that IUGR piglets had lower diversity of Bacteroidetes and Bacteroides in the jeunum at d 7, 21, and 28, Oscillibacter in the jejunum at d 21, and there was a positive correction between the Bacteroides and Oscillibacter abundances and the body weight of IUGR piglets [37]. A previous study also has indicated that the commensal bacteria such as *Lactobacillus* and *Streptococcus* were significantly decreased and the potential pathogens including *Fusobacterium* and *Campylobacter* were increased in the feces of IUGR piglets from d 7 to 21 of age, along with the altered concentrations of metabolites (e.g., fatty acid metabolism, bile acid biosynthesis and amino acid metabolism) [8]. Specially, qPCR outcomes revealed that the copy number of predominant *Lactobacillus* species like *L. salivarius* on d 7 and *L. amylovorus* on d 21 were significantly reduced in the colon of IUGR piglets [38]. Similarly, two trials conducted on rats and mice also reported that the cecocolic and fecal microbial composition were changed in IUGR infancy [39, 40], compared to their normal counterparts. In preterm infants, facultative anaerobes like *Enterococcus*, *Enterobacter*, *and Lactobacillus* spp., were prevalent, while amounts of strict anaerobes and advantageous intestinal microbiota such as *Bifidobacterium and Bacteroides* were uncommon [41, 42]. In addition, low diversity of intestinal microbiota and prevalence of pathogenic bacteria were usually present in the intestine of preterm infants, which embodies a typical example of dysbiosis [43, 44]. More remarkably, recent experiments identified an increased abundance of *Escherichia-Shigella* and a decreased abundance of *Clostridium_sensu_stricto_1* in IUGR piglets, which was closely associated with the alterations of cytokines (tumor necrosis factor-α, interleukin-6, interleukin-1β and interferon-γ,) and plasma metabolites in the first 12 h of life (unpublished data), suggesting early-life interactions between intestinal microbiota and the intestinal immune function in IUGR piglets.

The above results indicate that the IUGR piglets have an intestinal dysbiosis, which is associated with the alteration in intestinal adaptation and microbial composition during the neonatal period.

### Maternal imprinting on intestinal microbiota of the IUGR piglets by vertical microbial transition during gestation

It is widely accepted that the microbiota in neonates was firstly established at birth, along with the exposure to microbes existing in the maternal vaginal canal during natural labor or the maternal skin during a cesarean. However, the conventional idea of ‘sterile womb’ has been questioned with an increasing attention of vertical microbial transition from mother to offspring [45]. Increased number of scientific studies from healthy full-term women have shown that there was bacterial DNA in placenta [46], amniotic fluid [47], umbilical cord blood [48], and meconium [49, 50]. Also, a recent experiment by meta-genomic analysis revealed that the human utero including cervical canal and peritoneal fluid contains microbiota [51]. However, some opposite arguments have been put forward, mainly because the research results above could not exclude the contamination [52]. Correspondingly, some suggestions to reduce the impact of contaminations in low biomass microbial studies have been made [52, 53]. All these results remind us that the effects of maternal imprinting on intestinal microbiota of the neonates might start from the intrauterine environments, but whether the colonization of intestinal microbiota happened in fetal stage requires more work to get verification.

It is clear that the fetuses absorb the nutrients from the umbilical cord vein during their fetal stage. Consequently, the early microbial colonization in neonatal intestine is possibly influenced by the microbial metabolites in uterus. A study in sows found that the microbiota community in umbilical cord vein, ultimately, impacted the microbiota and fermentative end-products profile including short-chain fatty acids and branched-chain fatty acids of the neonatal piglets [54]. In humans, the relative richness of dominant phylum such as Firmicutes in placenta was significantly lower in the IUGR neonates [55]. Similarly, another study reported that the reduced microbial richness of placenta was accompanied with spontaneous preterm neonates [46]. Above two outcomes in human revealed that the close associations of the decreased placental microbiome with IUGR neonates. However, the effects of microbiota from the intrauterine environment on IUGR progeny are scant. More clinical trials and experimental animal studies are required to explore it further.

### Maternal imprinting on intestinal microbiota of the IUGR piglets during the perinatal and lactation period

Besides intrauterine environment during gestation, some other factors including delivery mode, gestational ages at delivery, as well as the feeding patterns and environmental factors during lactating period could also affect the microbiota colonization of the neonatal IUGR piglets [56].

The delivery mode could be one of the important drivers for establishment of the intestinal microbiota in neonates [57]. Compared to the caesarean-delivered piglets, vaginally-delivered piglets had higher bacterial densities including *Bacteroides*, *Prevotella* at d 7 and *Clostridium* XIVa at d 14, which was consistent with the relatively abundant *Bacteroides* in vaginal microflora of the healthy sows [58]. At the same time, the vaginally-delivered piglets had higher propionate in ileum and butyrate in the ascending colon [59], which could be used as energy sources and believed to be health-enhancing for host [60, 61]. Therefore, maternal delivery mode might be regarded as a possible factor for affecting early-life microbial structure of neonatal IUGR piglets.

Maternal gestational age at delivery is also an important variable contribution to the preterm births. By comparing the preterm and full-term piglets, Kamal et al. [62] found that colonization of the dominant bacteria, Enterobacteriaceae, at d 5 was delayed in preterm piglets. Similar reports for fecal microbial differences between human preterm and full-term neonates also suggested that the preterm neonates had delayed gut colonization of commensal anaerobe microbes and increased levels of pathogenic microorganisms [42, 63, 64].

The maternal impacts on the neonatal intestinal microbiota continue with lactation. The different effects between nursing and other feeding patterns such as milk replacer or compound feed on the intestinal microbiota of neonatal piglets have been reported [65, 66]. Compared to the sow-reared piglets, relative abundance of the *Lactobacillus* and *Escherichia* in colon of the neonatal piglets with commercial milk-replacer was notably decreased [67]. Feeding formula could predispose the piglets to necrotizing enterocolitis (NEC), and to be prone to *Clostridium perfringens* infection [68, 69]. Likewise, the changed microbial composition and enhanced concentrations of short-chain fatty acids in response to early milk-feeding in neonatal piglets have also been revealed by others [70, 71]. So, we can see the important role of sow’s milk in colonization of the neonatal intestinal microbiota. One of the studies has showed that the bacteria populations in milk might be a source of intestinal bacteria [72].

In addition, environmental factors during lactation also show clear links with the intestinal microbiota of neonates. Lactating sows contain large amounts of bacteria and can be easily obtained by the nursing piglets. A study has demonstrated that the fecal microbial composition and function in neonatal piglets on d 1 were inclined to be analogous with those in sow’s milk and nipple surface [73]. Also, when the neonatal piglets were transferred from one sow to another, their intestinal microbial communities would be closer to the subsequent nursing sow’s [74, 75]. Since the variations in rearing environment could be complex, more trials are required to determine the corresponding variables and their contributions to the colonization of intestinal microbiota during suckling piglets.

### Maternal nutritional intervention during lactation to improve colonization of the advantageous intestinal microbiota in neonatal IUGR piglets

It is obvious that the lactating sows require a diet that could supply enough energy and nutrients to support their individual maintainence and also the growth of their offspring through milk production. As an important nutritional source, colostrum and milk could influence the establishment and succession of intestinal microbiota in neonates [76]. Thus, an enhanced maternal microbiota might provide advantageous microbes for either direct colonization or for indirect influence on the succession of indigenous intestinal microbiota in neonates. There were many studies showing the supplementation of probiotics and prebiotics for sows that could improve the colonization of beneficial intestinal microbiota in neonatal piglets [77, 78]. Here we mainly focused on discussing the effects and advancements of breastfeeding, probiotics and prebiotics supplementation for improving the intestinal health and colonization of intestinal microbiota in IUGR piglets.

#### Breastfeeding

Milk is the first diet source of neonates. It has a variety of biological functions, including supply of nutrients, protective Ig, antimicrobial and anti-inflammatory factors, which could enhance the early GIT development [79, 80]. It is worth noting that pigs have a very restricted transference of maternal Ig through the placenta, and thus, colostrum is the only source of Ig for neonatal piglets [81, 82]. Previous data confirmed that the multiple bioactivities (mainly Ig) of colostrum could regulate the innate immune reaction of intestinal epithelial cells [83]. Of note, IUGR piglets had delayed and lower amounts of colostrum intake than the NBW piglets [84, 85], which might be an important reason for intestinal immune deficiency and impairment. A recent research has evidenced that colostrum feeding partially ameliorated the inferior status of jejunal mucosa in IUGR piglets [86], thus probably leading to the change of establishment and composition of their intestinal microbiota. Moreover, 16S rRNA sequencing outcomes noted that the diversity of sow milk microbiota altered markedly in colostrum but remains relatively stable in transitional milk and mature milk [87], these results are line with the results of Liu et al. [88]. Cross-fostering could be a helpful practice to promote the quantity of colostrum received by the IUGR piglets. Maradiaga et al. has proposed that cross-fostering did not influence microbial composition present in the piglets GIT, but there was a notably correction between microbial communities of maternal colostrum and feces of piglets [89].

In addition, there is growing data suggesting that breastfeeding is one of the most key determinants of neonatal intestinal colonization. Not only because of the abundant bacterial communities in milk [90], but because a rich and natural source of oligosaccharide (OS) that regarded as a prebiotic activity, although the origin composition of milk microbita and OS are relatively complex and not completely illuminated [91]. Results in preterm infants have pointed out human milk OS could enhance the initial bacterial diversity and decrease the occurrence of NEC [92, 93]. By comparing the characterization of porcine milk OS and their relation to the fecal microbiota, Salcedo et al. investigated that fucose-consuming bacterial taxa in the intestinal microbiota of piglets were qualitatively but not quantitatively different between suckling and weaning stages [94], indicating that the composition and structure of milk OS may be important in shaping the intestinal microbiota of piglets. Besides, from the aspects of vertical transfer of sow’s microbiota, recent data suggested that the microbes from teats or the milk canal and feces are primarily responsible for the initial colonization of neonatal intestinal microbiota [88]. So, Further studies detecting the composition and function of milk-associated OS, might be useful to the development of intestinal health of IUGR piglets.

#### Probiotics/prebiotics

Most often, probiotics with the highest positive effects on human and animal GIT health are believed to be *Lactobacillus* species, *Bifidobacterium* species, *Enterococcus faecium* strains (commonly habitat in gastrointestinal tract), *Bacillus* genus spores (commonly habitat in soil), *Saccharomyces cerevisiae* yeast strains, etc. [95]. In the model of preterm/very low birth weight infants, a growing number of data has revealed that maternal supplementation with probiotic bacteria could reduce the occurrence of NEC and improve the infant weight [96, 97]. A meta-analysis even evidenced that combination of probiotics seems to be more effective than a single probiotics in preventing NEC and mortality of preterm/very LBW infants [98]. For example, supplementing combined probiotic milk (*Lactobacillus rhamnosus* GG, *Lacidophilus* La-5, and *Bifidobacterium animalis* subsp. *Lactis* Bb-12) from 36 weeks of gestation up to 3 months postnatally, can notably elevate the relative abundance of administrated probiotics in mothers, but only the *Lactobacillus rhamnosus* GG bacteria colonized the infant at 10 days and at 3 months of age [99], which indicates that different probiotic bacteria appear to have different ability to transfer from the mother to their offspring, thereby having different effects on their progeny. Either administrating the *Bacillus* or *Enterococcus faecium* probiotic strain to sows significantly increased the counts and quantity of *Lactobacillus* species [100, 101], and decreased the *Clostridium* spp. in the feces of neonatal piglets [101, 102]. Also, oral supplementation of nine microbial species supplements to lactating sows made the *Clostridium* cluster IV and subcluster XIVa particularly increased in their weaned piglets [103]. Above information suggests that probiotics or their combination during lactation period might be a potential intervention for reshaping the intestinal microbiota in IUGR piglets, but attention should be paid to the type of probiotics.

Prebiotics can selectively provoke the beneficial growth or activity of advantageous bacteria [104]. Previous results have proposed that inulin addition during the gestation and lactation can enhance the numbers of enterococci in sows. Also, a higher level of enterococci were detected in the cecal content of the suckling piglets [105]. Feeding diets with high-resistant starch (amylose corn) to sows from gestation to lactation increased milk nutrients probably via changing maternal intestinal microbiota composition, thus improving growth trait of offspring [78]. These emphasized that maternal prebiotics supplementation might be a useful method to modulate the intestinal microbiota and health in IUGR piglets. On the contrary, supplementation of resistant starch (pea starch) during gestation and lactation affected the fecal microbiota of the sows, but not that of their progeny, and neither the body weight or frequency of diarrhea of the piglets [106]. This attributes to the characteristics of the different types of fibers. On the whole, because of the complexity and diversity of fiber types, little understanding on corresponding metabolic condition of sows and the microbial imprinting on next generation exist nowadays. Therefore, relevant work needs to be pushed forward.

## Conclusion

In conclusion, intestinal development and microbiota colonization in the piglets were negatively affected by IUGR, due to imbalanced allocation of limited nutrients among fetuses during gestation. This is connected with the maternal microbial influences during gestation, at delivery or during lactation, and even at the pre-gestational stage through imprinting of the oocyte maturation. Maternal nutritional interventions with breastfeeding, probiotics or prebiotics could also help the colonization of advantageous intestinal microbiota in IUGR piglets.

## Data Availability

Not applicable.

## Abbreviations

GIT: Gastrointestinal tract
Ig: Immune globulin
IUGR: Intrauterine growth restriction
NBW: Normal birth weight
NEC: Necrotizing enterocolitis
OS: Oligosaccharide
